# Genome-Wide Expression Profiling Deciphers Host Responses Altered during Dengue Shock Syndrome and Reveals the Role of Innate Immunity in Severe Dengue

**DOI:** 10.1371/journal.pone.0011671

**Published:** 2010-07-20

**Authors:** Stéphanie Devignot, Cédric Sapet, Veasna Duong, Aurélie Bergon, Pascal Rihet, Sivuth Ong, Patrich T. Lorn, Norith Chroeung, Sina Ngeav, Hugues J. Tolou, Philippe Buchy, Patricia Couissinier-Paris

**Affiliations:** 1 French Army Biomedical Research Institute (Institut de recherche biomédicale des armées, IRBA), Antenne de Marseille-IMTSSA; Unité de Virologie, Marseille, France; 2 Institut Pasteur in Cambodia, Department of Virology, Phnom Penh, Cambodia; 3 TAGC-INSERM U928, Marseille, France; 4 Kampong Cham Provincial Hospital, Kampong Cham, Cambodia; Fundação Oswaldo Cruz, Brazil

## Abstract

**Background:**

Deciphering host responses contributing to dengue shock syndrome (DSS), the life-threatening form of acute viral dengue infections, is required to improve both the differential prognosis and the treatments provided to DSS patients, a challenge for clinicians.

**Methodology/Principal Findings:**

Based on a prospective study, we analyzed the genome-wide expression profiles of whole blood cells from 48 matched Cambodian children: 19 progressed to DSS while 16 and 13 presented respectively classical dengue fever (DF) or dengue hemorrhagic fever grades I/II (DHF). Using multi-way analysis of variance (ANOVA) and adjustment of p-values to control the False Discovery Rate (FDR<10%), we identified a signature of 2959 genes differentiating DSS patients from both DF and DHF, and showed a strong association of this DSS-gene signature with the dengue disease phenotype. Using a combined approach to analyse the molecular patterns associated with the DSS-gene signature, we provide an integrative overview of the transcriptional responses altered in DSS children. In particular, we show that the transcriptome of DSS children blood cells is characterized by a decreased abundance of transcripts related to T and NK lymphocyte responses and by an increased abundance of anti-inflammatory and repair/remodeling transcripts. We also show that unexpected pro-inflammatory gene patterns at the interface between innate immunity, inflammation and host lipid metabolism, known to play pathogenic roles in acute and chronic inflammatory diseases associated with systemic vascular dysfunction, are transcriptionnally active in the blood cells of DSS children.

**Conclusions/Significance:**

We provide a global while non exhaustive overview of the molecular mechanisms altered in of DSS children and suggest how they may interact to lead to final vascular homeostasis breakdown. We suggest that some mechanisms identified should be considered putative therapeutic targets or biomarkers of progression to DSS.

## Introduction

Acute dengue virus infections are a major public health problem for many tropical and sub-tropical countries and an increasing risk for the worldwide population [1]. Symptomatic infections occur under a spectrum of diseases ranging from classical dengue fever (DF) to the most severe life-threatening dengue shock syndrome (DSS), a leading cause of childhood hospitalisation and death in endemic countries with limited health resources [1], [2].

DSS is regarded as a vascular disease involving a complex interplay between virus, whole blood cells and microvascular territories [3], [4], and thought to result largely from an aberrant host response to infection. As for other major systemic diseases, a detrimental cytokine storm is thought to be central to the systemic microcirculatory failure and massive plasma leakage leading to cardiovascular decompensation characterizing DSS [5]. However, controversies exist regarding the nature of pathogenic host immune responses supporting this life-threatening syndrome [6]–[8]. Indeed, reactivation of cross-reactive memory T lymphocytes and increased infection of monocytes mediated by cross-reactive antibodies acquired during previous infections by distinct dengue virus serotypes, are the main hypothetic mechanisms proposed to explain the putative cytokine storm leading to plasma leakage [5], [9]. However, those hypothesis fail to explain the occurrence of DSS in patients having primary dengue infection and their relevance to the pathophysiology of DSS disease is discussed [8], [10].

Efforts to identify soluble biomarkers of severe dengue differentiating uncomplicated dengue infections from severe ones has led to the identification of a diversity of cytokines, chemokines, endothelial agonists or soluble endothelial molecules [11]–[18]. However, discrepancies in definition of dengue severity, variability in patients cohorts characteristics, as well as in techniques and markers investigated, have impaired the identification of reliable sets of DSS biomarkers and the possibility to get a global overview of biological markers altered during DSS.

Understanding the molecular basis of DSS and identifying relevant DSS biomarkers thus remains a major challenge [5], [6]. Indeed, DSS occurs by the end of the acute infection in only a fraction of dengue-infected patients and current severity criteria, based on the 1997 World Health Organization (WHO) classification of dengue severity, fail to predict a significant proportion of patients who progress to life-threatening DSS [19]–[21]. Attempting to decipher molecular mechanisms underlying DSS by analyzing circulating whole blood cell genome-wide expression profiles is a relevant approach regarding the study of other systemic inflammatory syndromes, where a cognate cross-talk between endothelial vascular cells and blood cells occurs [22]–[24]. Whole blood represent a highly informative while complex cellular sample, that may reflect host pathophysiological responses ongoing at the time of blood sampling [22]. Furthermore, whole blood cells are easy to collect and store during field studies on large cohorts, reducing samples volumes required and limiting technical bias due to cell purification. However, due to the high cellular complexity of whole blood cells samples, whole gene expression patterns should be carefully analyzed and deciphered to allow returning to an integrative view of the molecular mechanisms altered during the pathophysiological process studied [25].

Such a bench-to-bedside medical research has gained more and more interest in the recent years. Indeed, it allowed improving the understanding of pathophysiological processes underlying systemic critical illnesses such as sterile and non sterile systemic inflammatory responses syndromes (SIRS), allowing the identification of relevant disease biomarkers and of new putative therapeutic targets [22]–[24], [26].

Genome-wide expression studies aimed at deciphering molecular responses altered in the whole blood cells of adults [27] and children DSS patients [28] have been implemented recently by colleagues. They reported a decreased IFN type I-induced response and a benign transcriptional response at the time of cardiovascular decompensation [27], [28], but failed in identifying biological pathways relevant to DSS pathophysiology and particularly inflammatory ones that could sustain microvascular dysfunction [28].

We report here the results of a prospective study comparing the whole blood genome-wide expression profiles of 48 matched Cambodian children recruited during the huge 2007 dengue outbreak who presented with classical dengue fever (DF), dengue hemorrhagic fever grades I/II (DHF) or dengue shock syndrome (DSS), according to the 1997 WHO classification of dengue severity [29]. Based on careful study design and statistic treatment of microarrays data, we identified a large and highly relevant gene signature of DSS never reported before, that discriminates DSS children from paediatric patients with DF or DHF grades I/II, who did not present severe clinical complications. Using an integrative analysis of the gene patterns altered in DSS children, we deciphered part of the complex interactive molecular processes occurring during DSS, highlighting similarities between DSS and other major inflammatory processes. Finally, we identified unexpected pro-inflammatory innate immune responses activated in the whole blood cells of DSS children that may play a major role in DSS pathophysiology. The implications of present findings to the improvement of DSS prognosis and treatment are discussed.

## Materials and Methods

### Ethics statement

The global study and all protocols presented here were approved by the national Cambodian ethical committee. Written informed consent was obtained from the legal guardians of each child. To ensure strict anonymity regarding the patients, samples were encoded as PLxxx (Plasma Leakage).

### Patients and clinical data

Inclusion criteria retained were: age (1 to 15 years old); positive diagnosis of acute dengue infection assessed by different methods; absence of known chronic inflammatory disease or ongoing acute co-infection at the time of inclusion.

An eligible cohort of 83 dengue-infected children hospitalised at the Kampong Cham provincial hospital, Cambodia, was prospectively enrolled from July to September 2007 during the huge 2007 dengue outbreak in Cambodia, characterized by a high number of DSS cases.

Children diagnosed with acute dengue infections were classified at admission as classical dengue fever (DF), dengue hemorrhagic fever (DHF) or dengue shock syndrome (DSS) based on the 1997 WHO criteria [29]. Clinical and biological follow-up was done daily for each hospitalised patient. DSS patients were admitted to hospital intensive care unit where they received appropriate fluid resuscitation and were monitored for vital parameters. Children who required blood transfusion were not included in the study.

To increase the probability to identify gene signatures specific of DSS, we chose to include only symptomatic dengue-infected classified DF, DHF and DSS, but no healthy or non-dengue children in the present study. This is based on the rationale that comparing DF, DHF and DSS patients together should improve the probability to identify a DSS-specific gene signature, while including an external non dengue control group should increase the probability to identify a general dengue-related signature but should be less powerful at identifying a signature of severe dengue disease.

DF, DHF and DSS patients whole blood samples selected for the present study corresponded to comparable duration of illness after onset of fever: all were collected within a window of time comprised between 3 days and 7 days after onset of fever (being considered day 0). For most DSS patients, this generally corresponded to the day of cardiovascular decompensation (shock) or the day after, except for 3 (PL017, PL033, PL047) and 2 DSS (PL005, PL101) for whom blood was collected respectively 2 and 3 days after onset of shock.

Patients' samples selected for the present study were also carefully matched for age, gender, viral serotype (when identified) and immunological status (primary or secondary, according to reference assays described in diagnosis methods) towards dengue infection.

Diagnosis assays carried out as described thereafter, indicated that about 90% of all dengue-infected children had secondary infection.

### Dengue diagnosis and immunological status

All diagnosis assays were carried out at the Institut Pasteur in Cambodia, the National Reference Center for arboviral diseases in Cambodia. IgM capture ELISA and Hemagglutination-inhibition were performed on paired sera collected at admission and at discharge, and systematically tested for both dengue and Japanese Encephalitis virus, another flavivirus endemic in Cambodia, as described previously [30]. Virus isolation was carried out on earliest serum samples by inoculating permissive C6/36 and VERO E6 cells, followed by serotype-specific immunofluorescence [30]. Viral RNA was detected in specimens collected at early stage of the disease using a nested RT-PCR [31]. Primary or anamnestic “secondary” antibody response indicating previous infections by dengue viruses was determined from paired serum samples by hemagglutination-inhibition assay. Interpretation followed WHO recommendations [29].

### Blood sample preparation

Whole blood samples (2.5 ml) were collected on PAXgene™ Tubes (PreAnalytiX™) further stored at −80°C, before being sent to France in dry ice. Extraction of series of 24 matched samples (DF, DHF and DSS) was done using PAXgene™ Blood RNA kits (PreAnalytiX™) rapidly after collection. Purified total RNAs kept at −80°C were processed for hybridization on genome-wide DNA microarrays within one month.

### cRNAs preparation and microarrays hybridization

All RNAs were checked for integrity using the 2100 BioAnalyzer (Agilent Technologies) and quantified using a ND-1000 spectrophotometer (NanoDrop Technologies). Cyanine-3-labeled cRNA was generated from 0.3 µg of RNA using the One-Color Low RNA Input Linear Amplification kit (Agilent) according to the manufacturer's instructions, followed by purification on RNAeasy column (QIAGEN). All amplified cRNAs were checked for dye incorporation, cRNA yield and amplification profile. Only those fitting all quality criteria were fragmented for further hybridization on microarrays. Samples from DF, DHF and DSS patients were then carefully matched and hybridized onto Agilent Whole Human Genome (4×44K) Oligo Microarrays (G4112F). Microarrays were scanned using an Agilent DNA microarray scanner G2505B.

### Microarray data analysis

All microarray data is MIAME compliant and the raw and normalized data have been deposited in the MIAME compliant database Gene Expression Omnibus [32] (GEO Series accession number GSE17924 http://www.ncbi.nlm.nih.gov/geo/query/acc.cgi?acc=GSE17924).

Individual microarray quality was evaluated based on QC report, pair-wise MA-plots, and box plots. Intra-array normalization of raw signals from the 48 microarrays was done using Feature Extraction software 9.1.3.1 (Agilent). Microarrays normalized data were further exported into the Limma package [33], for inter-array normalization using the quantile method [34].

Statistical analysis were was performed using the TIGR MeV (MultiExperiment Viewer) v 4.4 software (http://www.tm4.org/mev.html), and the GeneANOVA program [35]. Multi-way ANOVA model was implemented: first, to identify differentially regulated genes when accounting for the multiple sources of variation in the microrray experiment; second, to evaluate the effect of the main variable, disease phenotype, relatively to that of other putative confounding variables such age, gender, duration of illness or microarray technical variability (independent extractions or hybridizations). Local ANOVA further determined the contribution of each covariate on the expression level of each gene. Multiple test correction was further carried out using the false discovery rate (FDR) method [36]. Cluster [37] and Tree View softwares [38] were used for unsupervised hierarchical clustering. Iterative SVM (Support Vector Machine) method associated with leave-one-out cross-validation [39] was used to assess the robustness of DSS-gene signature.

### Real-time PCR validation of genes over and under-expressed in DSS patients

Briefly, total RNA extracted from whole blood samples was reverse-transcripted using the High Capacity cDNA RT kit (Applied Biosystems Inc) and random primers. Real-time PCR were carried out using the FastStart Universal Probe Master (ROX) (Roche) and real-time PCR primers designed using the Universal Probe Library (UPL) Assay Design Center (Roche). Amplification products were run on an ABI-PRISM 7900HT (Applied Biosystems). Cycle threshold Ct values were automatically calculated and value obtained for each gene amplified was normalized by subtracting the Ct corresponding to amplification of the HPRT1 gene (ΔCt) for the same sample. Correlation between ΔCt values obtained by real-time PCR and corresponding expression values from microarrays was estimated using Spearman correlation coefficient.

### Comprehensive overview of functional patterns altered during DSS

Bio-informatics-based analysis using the demonstration version 7.1 of Ingenuity Pathway Analysis software (IPA; Ingenuity® Systems, www.ingenuity.com) associated with manual and litterature-based analysis was carried out to identify the most relevant functional processes associated with the identified DSS gene signature. This was done by combining most informative canonical pathways identified using IPA, genes having the strongest association with the disease phenotype based on ANOVA analysis, and similarities to molecular patterns altered in other systemic inflammatory processes associated with endothelial dysfunction.

## Results

### Patient characteristics

To identify gene patterns specifically altered in DSS patients, we compared three groups of carefully matched paediatric patients representing the main clinical forms of symptomatic dengue infections DF (n = 16), DHF (n = 13) and DSS (n = 19), according to the 1997 WHO classification criteria of dengue severity [29]. Altogether, DF, DHF and DSS represent different subtypes of the disease phenotype variable, further considered in this study. The clinical characteristics and values of haematological parameters are presented in table 1 (median values from each patient group) and table S1 (individual values from each of the 48 patients included). Supportive treatment provided to DSS patients are mentioned. As indicated, DSS children had significant lower relative neutrophil counts (median values / DF: 3900; DHF: 3950; DSS: 2500; p-value: 0.03; Kruskal Wallis test).

**Table 1:** Clinical and biological characteristics of DF, DHF and DSS patient groups at the time of hospital admission.

	DF (*n* = 16)	DHF (*n* = 13)	DSS (*n* = 19)
Patients characteristics			
gender, male *n* (%)	7 (43%)	4 (31%)	7 (37%)
age, median (IQR), years	8 (4–9)	7 (5–8)	8 (7–9)
weight, median (IQR), kg	18 (13–20)	15 (14–18)	19 (15–23)
hospital admission, median (IQR), day after onset of fever (D0)	2 (1–3)	2 (2–3)	4 (3–4)
Dengue status			
viral serotype, *n* (DENV-1/DENV-2/DENV-3/DENV-4/unknown)	4/2/8/1/1	1/1/10/1/0	1/1/10/0/7
immunological status, secondary infections, *n* (%)	14 (88%)	12 (92%)	18 (95%)
Clinical manifestations			
tourniquet test (pos/neg/not done) (%)	56%/44%/0%	54%/38%/8%	37%/32%/31%
hepatomegaly, *n* (%)	3 (19%)	6 (46%)	17 (89%)
gastro-intestinal bleeding, gingivorragy, hematemesis, melena, *n* (%)	0	1 (8%)	6 (32%)
Blood pressure			
heart frequency, median (IQR), pulse per minute	113 (100–124) (*n* = 14^a^)	120 (112–120)	Not perceptible (*n* = 15^a^)
pulse pressure, median (IQR ), mm Hg	40 (30–45)	30 (30–40)	15 (10–20) (*n* = 15^a^)
Haematological parameters			
thrombocytopenia (platelet count <100000/mm^3^), %	15% (*n* = 14^a^)	55% (*n* = 11^a^)	94% (*n* = 17^a^)
hematocrit, median (IQR), %	36.5 (35–39) (*n* = 14^a^)	39.75 (38–42) (*n* = 12^a^)	42.5 (38–45) (*n* = 18^a^)
hemoconcentration (hematocrit >20%), *n* (%)	1 (6%)	3 (23%)	17 (89%)
white blood cells, median (IQR), number/mm^3^	6600 (5500–9900) (*n* = 13^a^)	6450 (6200–7400) (*n* = 10^a^)	6900 (4800–6900) (*n* = 17^a^)
neutrophils, median (IQR), number/mm^3^	3900 (2900–7600) (*n* = 13^a^)	3950 (3500–4200) (*n* = 10^a^)	2500 (2200–3800)(*n* = 17^a^)
lymphocytes, median (IQR), number/mm^3^	1600 (1400–2100) (*n* = 13^a^)	1850 (1500–1900) (*n* = 10^a^)	2200 (1500–3200) (*n* = 17^a^)
Supportive medical care			
oxygen supplementation, *n* (%)	0	0	15 (79%)
perfusion of colloid (dextran 40), *n* (%)	0	0	14 (74%)
perfusion of human plasma, *n* (%)	0	0	8 (42%)

DENV, dengue virus; DF, dengue fever; DHF, dengue hemorrhagic fever; DSS, dengue shock syndrome; IQR, interquartile range; n, number.

a n = x : with x : number of patients for which the data is available.

### Unsupervised hierarchical clustering discriminates DSS children from DF/DHF ones, revealing a DSS-gene signature

Since microarray data analysis can be affected by a number of bias [40], we put a particular care on study design and analysed data from the 48 normalized microarrays using multi-way analysis of variance (ANOVA) [41]. Indeed ANOVA evaluates the statistical probability (p-value), for each individual gene, that a difference in expression between the three patient groups could have been observed by chance. This allows revealing genes that show even small but highly significant changes in expression, regarding the studied phenotype.

Based on ANOVA analysis, lists of genes differentially expressed between DF, DHF and DSS groups were generated using different false discovery rate (FDR) ranging from 0.05 up to 10%. Indeed, low FDR provide more stringent statistical filter while they reduce the number and thus the enrichment of genes differentially expressed. At the opposite, higher FDR, while statistically accepting a higher number of false positive genes, also provide larger and enriched gene lists that should be more informative when searching to identify molecular pathways. Based on this rationale, we chose to work using the gene list generated at FDR 10 after we verified by a different statistical method currently used for the analysis of microarrays data, SAM (Significant Analysis of Microarray) [33], that most significant genes were commonly found by the two types of analyses (data not shown). The gene list generated at FDR10 included 2959 genes differentially expressed between DF, DHF and DSS patients groups (Table S2).

The biological relevance of those differentially expressed genes was assessed using local ANOVA that allows evaluating the contribution of the main variable, disease phenotype, and that of other putative confounding variables related to patients (age, gender, day of blood sampling, viral serotype) and to technical steps (effect of independent RNA extractions, amplifications and hybridization) on variations of expression levels of those 2959 genes. This confirmed that the disease phenotype strongly influenced the variations of expression of the 2959 genes differentially expressed between the three patient groups, reinforcing the biological significance of this set of genes (Table S2).

**Figure 1:**
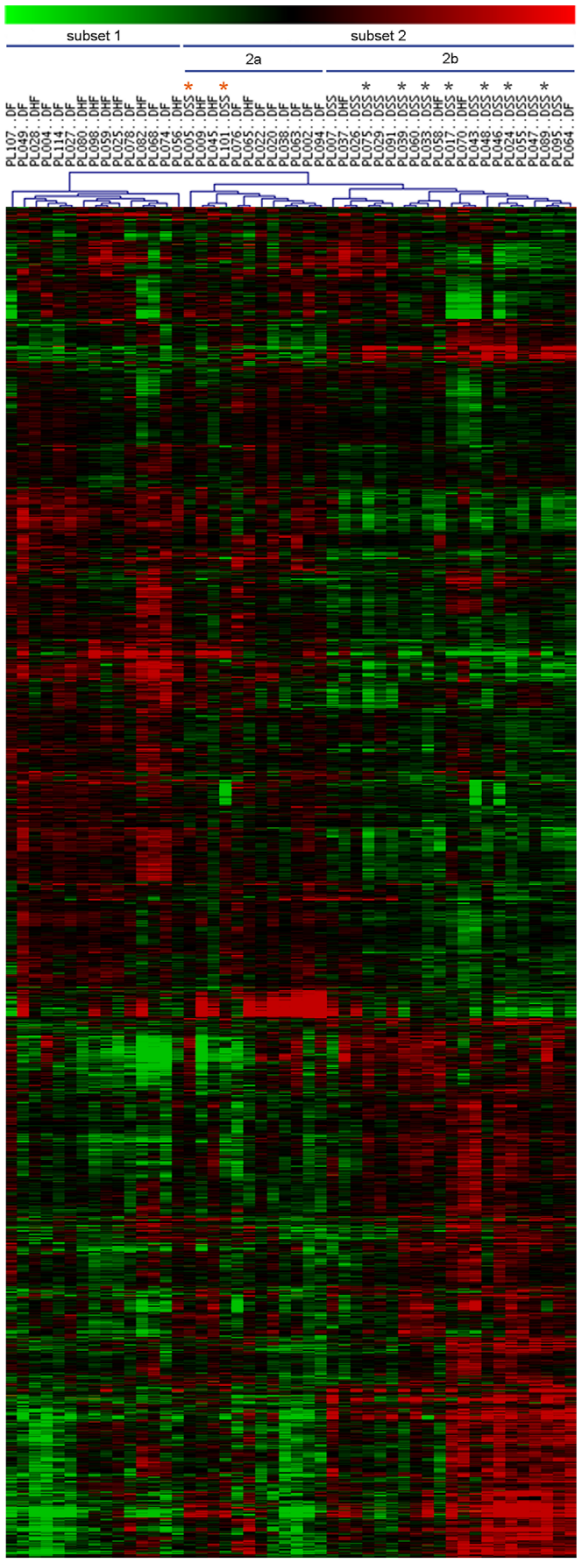
Unsupervised hierarchical clustering of whole blood cells expression profiles from the 48 dengue-infected children. The clustering is based on the 2959 gene list (3515 clones, detailed in Table S2) discriminating dengue fever (DF), dengue hemorrhagic fever (DHF) and dengue shock syndrome (DSS) patients. Each row represents a single transcript and each column represents a patient's sample. Color scale indicates the range of gene expression: black indicates median expression level, red greater expression, green lower expression. The 2 patient subsets identified are indicated. PLxxx, code relative to one patient. Black star: DSS patient sampled 3 days after shock. Orange star: Patients who received perfusion of human plasma before collection of blood samples.

Unsupervised hierarchical clustering based on the 2959 gene signature identified was then applied to the 48 children gene expression profiles. This allows clustering the patients whose gene expression profiles are the more similar independently of their disease phenotype subtype. As a result, the 48 patients expression profiles were organized in two major subsets (Figure 1): subset 1 (first dendrogram branch) includes both DF and DHF patients without distinction; subset 2 (second dendrogram branch) encompasses a sub-group (2a) of DF and DHF patients, and a distinct sub-group (2b) including 17 out of the 19 DSS patients, whatever they received or not plasma infusion, revealing a “DSS-gene signature” common to most DSS patients. Some few patients clustered however in unexpected subsets: two DSS patients (PL005, PL101) had gene expression profiles closer to those of the DF/DHF 2a subset, while one DF patient (PL064) and three DHF patients (PL037, PL058, PL070) gene expression profiles clustered within the DSS 2b subset.

We confirmed the robustness of the DSS-gene signature using the iterative Support Vector Machine (SVM) classifier learning method [39], which reclassified all the 19 DSS patients together.

To validate microarray data, we carried out real-time RT-PCR focusing on nine genes strongly associated with the DSS-gene signature, using 15 patients samples (five from each disease phenotype subtype: DF, DHF and DSS). Results obtained strongly correlate microarray data (Figure S1).

### DSS-gene signature analysis identifies a diversity of genes and canonical molecular pathways related to immunity, inflammation and host metabolism

Filtering genes from those having the highest to the lowest statistical association with the disease phenotype variable (Table S2), relying on results from multi-way and local ANOVA revealed that the individual genes having the strongest association with the DSS phenotype subtype are, for a large part, related to innate immunity, inflammation and host lipid metabolism, a finding confirmed when the whole 2959 genes of the DSS-gene signature were processed through the knowledge-based IPA software.

**Figure 2:**
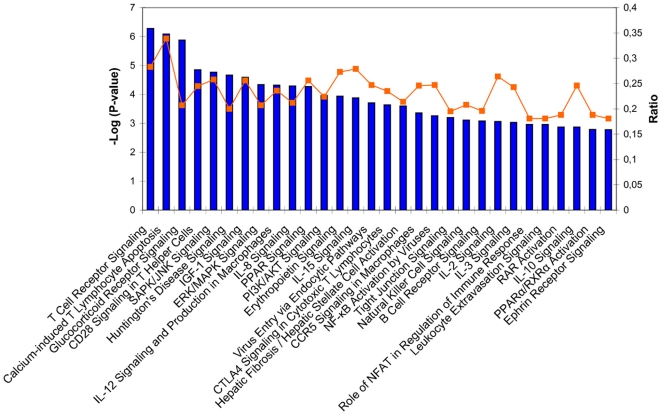
Top 30 canonical pathways identified from the DSS-gene signature using Ingenuity Pathway Analysis software. The significance of the association between data set and canonical pathway was estimated by the p-value (Fischer's exact test; left axis) and the ratio (right axis) of genes that maps to each canonical pathway.

**Figure 3:**
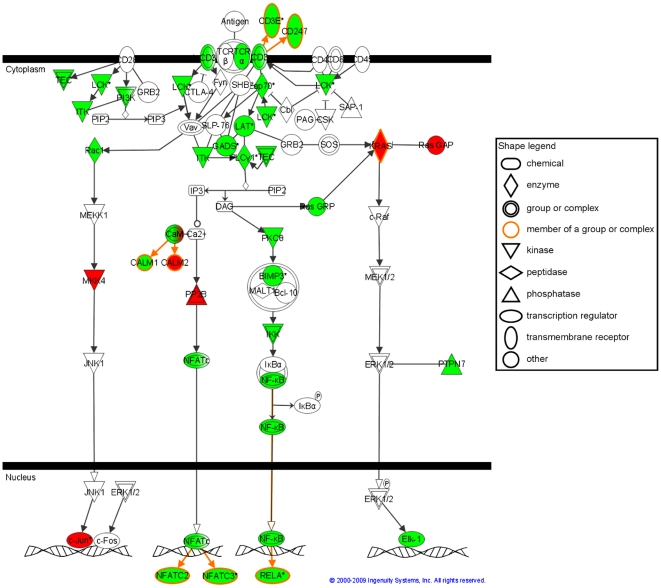
T Cell Receptor Signaling canonical pathway from Ingenuity Pathway Analysis. Genes in green and red are respectively under- and over-expressed in the DSS-gene signature. Genes in white are other genes present in the canonical pathway but absent from the DSS-gene signature. DSS: Dengue Shock Syndrome.

Indeed, IPA analysis identified that 163 canonical pathways were significantly associated with those genes (data not shown) with a large proportion of immune-related pathways in the first top 30 (Figure 2). In particular, several under-expressed but partially redundant signaling canonical pathways related to T lymphocyte activation were identified, of which the T cell receptor (TCR) signaling pathway (Figure 3), which has the strongest association with the DSS-gene signature. Interestingly, a number of metabolic pathways, and particularly of lipid-signaling pathways, were significantly represented among the 163 DSS-related canonical pathways.

When comparing our results to those of colleagues who reported gene or protein signatures associated with DSS, we identified some transcripts encoding proteins considered putative markers of severe dengue. This includes non exhaustively the acute phase pentraxin-related protein PTX3 [15], the anti-inflammatory IL-10 [11] or the pro-inflammatory IL-18 [12] cytokine transcripts that have increased abundance in the DSS-gene signature, while having intermediate to low statistical association with the disease phenotype variable according to the multi-way ANOVA (Table S2).

IFN type I-related transcripts, of which abundance was shown to be decreased in DSS patients by others [27], [28], [42], represented only a limited number of genes associated with the DSS-gene signature. This was confirmed by the IPA analysis that did not identify IFN type I-related pathways among those strongly associated with the DSS-gene signature (Figure 2).

### DSS is associated with impaired expression of T and NK cell-related genes but increased expression of anti-inflammatory and repair/remodeling transcriptional responses

Integrative analysis of the most significant individual genes and canonical pathways extended the finding that a large and diverse set of genes related to T but also to NK lymphocyte activity is under-expressed in DSS patients compared to DF and DHF counterparts (Table 2; non exhaustive list; individual p-values available in Table S2; and Figure 3). Those genes are critical to a number of T and NK-cell functions, including T and NK cell differentiation, receptor signaling, activation and proliferation, cytotoxic functions or recruitment of lymphocytes to peripheral tissues. Since lymphocyte counts did not differ between the DF, DHF and DSS children (p = 0.428; Kruskal Wallis test), we searched whether genes encoding factors regulating negatively T and NK functions were over-expressed in the DSS-gene signature. We identified that the two genes having the strongest association with the disease phenotype variable, encode two major immunomodulatory factors, the microsomal prostaglandin E synthase (*PTGES*/Agilent clone number A-24-P478940) and the complement regulatory protein CRIg/VSIG4, considered potent negative regulators of T and NK cell responses [43]–[45]. The decreased abundance of *NFkB* signal transduction-related transcripts (Table 2), already reported in DSS patients by others [46], might be related to impaired expression of T and NK cell-related genes.

**Table 2:** T lymphocytes and NK cells-related genes present in the DSS-gene signature.

Function	Genes	P-value	Var.^a^ (%)
Th1 differentiation	RUNX3, STAT4, TBX21	<0.00001 to 0.00242	25 to 42
Th2 differentiation	GATA3, STAT5A	0.00003 to 0.00225	17 to 32
Cytotoxic T lymphocyte functions	CTSW, PRF1	0.00005 to 0.00231	21 to 33
T lymphocyte activation	IL2RB, IL2RG	0.00014 to 0.00039	29 to 35
Cooperation with antigen-presenting cells	CD40LG	0.00105	21
Recruitment and interaction of T lymphocytes with endothelium	ITGAL, XCL1, XCL2	<0.00001 to 0.00214	20 to 33
Inhibitory NK cell receptors	KLRD1	0.00001	31
Activating NK cell receptors	NCR1, NCR3, CD160	<0.00001 to 0.00069	28 to 39
Cytotoxic molecules	GZMM	<0.00001	32
Receptors for NK cells homing to peripheral tissues	S1PR5	<0.00001	48
Differentiation factors of NK cells	FLT3LG, **IL15**, **IL17C**, KITLG	0.00088 to 0.00774	13 to 21
Suppression of T lymphocytes and NK cells response	**PTGES**, **VSIG4**	<0.00001	60 to 63
NFκB-related genes	**IRAK3**, TNIK, RELA, NFKBIB, TRAF1, TRAF2, TRAF6	0.00001 to 0.00506	9 to 31

HUGO gene names are indicated. When genes were represented by several clones on the microarray, p-value and variance medians were calculated. Genes in regular and bold are respectively under- and over-expressed in dengue shock syndrome patients.

a percentage of variance associated to disease phenotype.

Our analysis also revealed that DSS whole blood cells from children over-expressed an enriched pattern of anti-inflammatory and repair/tissue remodeling genes (Table 3; non exhaustive list; individual p-values available in Table S2). Over-expressed anti-inflammatory genes identified encode molecules with diverse functions: the anti-inflammatory cytokine IL-10, a putative marker of severe dengue [11], serine proteases and metalloprotease inhibitors, IL-1β cytokine decoy receptor, free heme scavenger molecules, or complement-regulating receptors. Repair and remodeling genes over-expressed in the DSS-gene signature also encode a diversity of molecules: the MMP-9, a matrix metalloprotease with key role in tissue remodeling and a candidate to dengue plasma leakage [47], the extracellular matrix molecules fibronectin, versican and collagens, the angiogenin and VEGF [17] endothelial agonists as well as the arginase 1 repair enzyme, which competes with the endothelial NOS (*NOS3*) for L-arginine biodisponibility [48].

**Table 3:** Anti-inflammatory, tissue remodeling and repair genes present in the DSS-gene signature.

Function	Gene Symbol	P-value	Var.^a^ (%)
Anti-inflammatory genes			
immunoregulatory molecules	**IL10**	0.00430	20
anti-proteases	**SERPINB2**, **SERPINB8**, **SERPINB10**, **SLPI**	<0.00001 to 0.00081	19 to 49
metalloproteinase inhibitor	**TIMP1**	0.00183	19
decoy receptor	**IL1R2**	0.00077	30
free-heme scavenger molecules	**CD163**, **HP**, **HMOX1**	<0.00001 to 0.00064	26 to 46
complement regulatory molecules	**CD55**, **VSIG4**	<0.00001 to 0.00096	24 to 60
Tissue remodeling and repair genes			
metallopeptidase	**MMP9**	0.00001	33
extracellular matrix components	**COL1A2** b, **COL8A2** b, **COL14A1** b, **COL17A1** b, **FN1** b, **SDC1** b, **VCAN** b	<0.00001 to 0.00309	18 to 34
pro-angiogenic factors	**ANG**, **VEGFA**	0.00004 to 0.00236	25 to 30
others	**ARG1**, NOS3	<0.00001 to 0.00054	18 to 44

HUGO gene names are indicated. When genes were represented by several clones on the microarray, p-value and variance medians were calculated. Genes in regular and bold are respectively under- and over-expressed in dengue shock syndrome patients.

a percentage of variance associated to disease phenotype.

b Danger-associated molecular pattern (DAMP) activity.

Thus, DSS children whole blood cells have a global decreased abundance of T and NK cell-related transcripts but an increased abundance of anti-inflammatory and repair/remodeling transcripts at the time of cardiovascular decompensation.

### Pro-inflammatory innate defense and host-lipid metabolism-related transcriptional responses are activated in DSS children

When searching for pro-inflammatory gene patterns that may be relevant to DSS pathophysiology and particularly to systemic inflammation and vascular dysfunction, we identified three major pro-inflammatory gene patterns. Interestingly, all are related to innate defense and host lipid metabolism, and considered major pathogenic mechanisms in other systemic inflammatory diseases.

As shown in table 4 (non exhaustive list; individual p-values available in Table S2), the first one is defined by a set of over-expressed genes strongly associated with the disease phenotype subtype DSS that encode highly pro-inflammatory microbicidal peptides and enzymes. This pattern includes non exhaustively the alpha defensins DEFA1, DEFA3 and DEFA4, the cathelicidin (CAMP) and lactoferrin (LTF) peptides, the neutrophil enzymes myeloperoxydase (MPO), neutrophil RNASE2, RNASE3, cathepsin G and neutrophil elastase (ELANE). Transcripts encoding the potent pro-inflammatory calgranulins proteins S100A8/9 and S100A12, characteristic of granulocyte neutrophil activity [51] and involved in a diversity of inflammatory diseases [56], as well as the granulocyte-related metalloprotease MMP8 are also over-expressed. Increased abundance of those transcripts cannot be explained by increase in granulocyte count, since DSS patients have lower relative granulocyte counts than DF and DHF counterparts (median values/DF: 3900; DHF: 3950; DSS: 2500; p-value: 0.03; Kruskal Wallis test), thus reflecting more likely cellular activation. Altogether those results show that a transcriptional pattern of innate defense genes is activated in the whole blood of DSS children.

**Table 4:** Pro-inflammatory innate immunity-related genes present in the DSS-gene signature.

Function	Gene Symbol	P-value	Var.^a^ (%)	Main cellular origin	Ref
microbicidal peptides	**DEFA1** b, **DEFA3** b, **DEFA4** b	<0.00001 to 0.00007	0.25 to 0.44	PMN neutro, EpC	[49], [50]
	**CAMP** b	<0.00001	0.34	PMN neutro, Mo, mast cells, EpC	[49], [50]
	**LTF** b	<0.00001	0.41	PMN neutro, inflammed EpC	[50]
calgranulin proteins	**S100A8** b, **S100A9** b	<0.00001 to 0.00014	0.18 to 0.38	PMN neutro, Mo/Mac	[51], [52]
	**S100A12** b	<0.00001	0.33	PMN neutro	[51]
granulocyte enzymes	**RNASE2** b	0.00017	0.25	Mo/Mac, Eo, EpC, PMN neutro	[53]
	**MPO** b	0.00024	0.25	PMN neutro, Mo, subtypes of tissue Mac	[50]
	**RNASE3** b	<0.00001	0.29	Eo, Mo, PMN neutro	[54]
	**MMP8**	<0.00001	0.49	PMN neutro	[50]
	**CTSG**	<0.00001	0.36	PMN neutro	[50]
	**ELANE**	<0.00001	0.39	PMN neutro	[50]
pro-inflammatory cytokines and related molecules	**IL18**	0.00052	0.21	Kupffer cells, activated Mac, Mo, DC, EpC	[55]
	IL18BP	0.00710	0.20	T cells, peripheral blood leukocytes, EC	[55]

HUGO gene names are indicated. When genes were represented by several clones on the microarray, p-value and variance medians were calculated. Genes in regular and bold are respectively under- and over-expressed in dengue shock syndrome patients. DC, dendritic cell; EC, endothelial cell; Eo, eosinophil; EpC, epithelial cell; Mac, macrophage, Mo, monocyte; PMN neutro, polymorphonuclear neutrophil; RAGE, receptor for advanced glycation end products.

a percentage of variance associated to disease phenotype.

b Danger-associated molecular pattern (DAMP) activity.

The second pro-inflammatory gene pattern identified is typical of altered homeostasis of cholesterol in monocytes/macrophages that characterises inflammatory lipid-laden monocytes/macrophages (lipid-laden Mo/Mac), a subtype of foam cells initiating vascular lesions in metabolic inflammatory diseases [57]–[59] (Table 5; non exhaustive list; individual p-values available in Table S2). Since the *PPARG* gene, which encodes a nuclear-lipid receptor involved in lipid signaling and lipid-homeostasis in inflammatory lipid-laden Mo/Mac [65], has a very strong association with the dengue disease phenotype, we searched whether other genes involved in cholesterol homeostasis in Mo/Mac had altered expression in the DSS-gene signature. Remarkably, we found a large lipid-laden Mo/Mac-related gene expression pattern characterized in particular by a higher abundance of transcripts encoding the key scavenger receptors of modified low density lipoproteins OLR-1, CD36 and MSR1, but a decreased abundance of transcripts encoding critical cholesterol transporters such as the NPC1 [66] or the ABCA1-like ABCA10 [86], which regulate the efflux of modified cholesterol from Mo/Mac. Other lipid-laden cells-related genes also have altered expression in the DSS-gene signature. In particular, the *PPARA* gene that negatively regulates the formation of lipid-laden Mo/Mac [87] has decreased abundance in DSS patients. At the opposite, transcripts encoding the chitinase 1, a marker of pro-inflammatory lipid-laden Mo/Mac [73], and the FABP4, SOCS6, RETN and IRS2 proteins involved in lipid-laden Mo/Mac-induced insulin-resistance and compensatory response [68]–[72], have all increased abundance, also strongly supporting a biological signature of foam cells. Interestingly, the *PCSK9* transcript, which encodes a secreted protein that decreases the recycling of LDL to the liver by inducing the degradation of liver LDL receptors [74], is also over-expressed in the DSS signature and highly associated with the disease phenotype.

**Table 5:** Pro-inflammatory lipid-related genes present in the DSS-gene signature.

Function	Gene Symbol	P-value	Var.^a^ (%)	Disease	Ref
Lipid-laden Mo/Mac-related genes					
scavenger receptors of modified LDL in Mo/Mac	**OLR1**, **CD36**, **MSR1**	<0.00001 to 0.00013	0.21 to 0.32	metabolic diseases	[60]–[64]
lipid nuclear receptor/signalisation by lipids	**PPARG**, PPARA	0.00007 to 0.00732	0.21 to 0.34	metabolic diseases	[65]
efflux of modified cholesterol from Mo/Mac	NPC1	0.00005	0.32	Niemann-Pick disease, atherosclerosis	[66], [67]
	ABCA10	0.00016	0.14	none	
migrating Mo/resident Mac chemokine receptors	**CCR2**, CX3CR1	0.00001 to 0.00099	0.22 to 0.40	atherosclerosis	[57]
other lipid-laden-related Mo/Mac genes	**FABP4**, **SOCS6**, **RETN**, **IRS2**	<0.00001 to 0.00092	0.20 to 0.26	metabolic diseases	[68]–[72]
	**CHIT1**	<0.00001	0.48	Gaucher's disease, atherosclerosis	[73]
	**PCSK9**	0.00001	0.42	familial hypercholesterolemia	[74]
	**SPP1**	<0.00001	0.49	metabolic and inflammatory diseases	[75], [76]
anti-oxydant enzymes	LCAT, PAFAH2	0.00196 to 0.00461	21 to 26	metabolic diseases	[77]
Arachidonic acid pathway-related genes					
phospholipase	**PLA2G4A**	0.00003	0.21	rheumatoid arthritis	[78]
eicosanoid synthesis enzymes	**PTGES**, **LTA4H**, PTGDS, **TBXAS1**, PTGDR	<0.00001 to 0.00123	0.22 to 0.63	metabolic and inflammatory diseases, asthma, cancer	[79]–[82]
leukotrienes convertion enzyme	**MGST2**	0.00003	0.32	none	
leukotriene transporter	**SLCO2B1**	0.00010	0.31	asthma	[83]
lipid oxidation	**ALOX15B**	0.00011	0.33	atherosclerosis	[84]
cytochrome P450 superfamily enzymes	**CYP1B1**, CYP2U1, **CYP51A1**	<0.00001 to 0.00686	10 to 32	Vascular inflammation	[85]

HUGO gene names are indicated. When genes were represented by several clones on the microarray, p-value and variance medians were calculated. Genes in regular and bold are respectively under- and over-expressed in dengue shock syndrome patients.

a percentage of variance associated to disease phenotype.

Thus, a gene expression pattern similar to that characterizing lipid-laden monocytes, is activated in the whole blood cells of DSS children at the time of cardiovascular decompensation.

The third pro-inflammatory gene pattern associated with the DSS-gene signature is characteristic of the metabolic pro-inflammatory arachidonic-acid pathway, one of the lipid metabolic pathways identified through IPA. As shown in table 5, the gene encoding the upstream cytosolic phospholipase PLA2G4A, which is the initial rate-limiting enzyme that cleaves membrane phospholipids [78], is over-expressed. Similarly, most downstream key enzymes from the COX-2 and 5-LOX sub-pathways involved in the final synthesis, conversion and transport of inflammatory eicosanoids lipid mediators, are over-expressed. In particular, the transcript encoding the inducible microsomal prostaglandin E synthase PTGES that catalyzes the conversion of prostaglandin PGH2 to PGE2 in the COX-2 sub-pathway, and thought to play a pathogenic role in a number of inflammatory processes [88] is significantly increased and has the highest statistical association with the disease phenotype (62% of gene variance explained by the disease phenotype according to multi-way ANOVA). At the opposite the *PTGDS* transcript, which encodes the anti-inflammatory prostaglandin D2 synthase, has decreased abundance, a finding already reported in metabolic inflammatory processes [89]. Increased abundance of the transcript encoding the LTA4H enzyme that converts the LTA_4_ leukotriene to LTB_4_ reflects the activation of the 5-LOX sub-pathway. Finally, transcripts encoding the oxidative enzymes ALOX15B lipoxygenase [84] and cytochrome P450 epoxygenase family members [85], involved in the arachidonic acid metabolic pathway, are also significantly increased in the DSS-gene signature, also reflecting activation of those sub-pathways during DSS.

Thus, a transcriptional signature related to the lipid-related metabolic arachidonic acid pathway is activated in the whole blood cells of DSS children at the time of cardiovascular decompensation.

## Discussion

Numerous studies have addressed the pathophysiology of DSS, the more frequent and severe complication of dengue infections. Despite important findings, only partial understanding of the cellular and molecular processes that may support this life-threatening syndrome has been obtained, and we still lack a comprehensive overview of the complete figure of alterations that contribute to – or reflect – the setting-up of the shock syndrome. This could allow the improvement of patients' management and treatment, a major challenge for clinicians. We designed a study aimed at analysing the quasi-global transcriptome of whole blood cells from dengue paediatric patients, looking at every modification that could make sense to the understanding of the pathogenic process. The capacity of such an exhaustive approach to identify relevant host responses, of which unsuspected pathways has been demonstrated in other systemic inflammatory syndromes such as human sepsis or post-trauma sterile SIRS [23]–[25]. We compared the transcriptome of blood cells from DSS paediatric patients at time of shock to those of children classified as DF or DHF grades I/II [29] matched for important variables such as age, gender, immune status towards dengue infection (primary or secondary infection) and time of disease evolution after onset of fever. Our study has produced significant results, further discussed in the context of DSS pathophysiology.

First, we identify a transcriptional signature of the DSS, differentiating DSS from the other forms of dengue infection and characterizing DSS as a unique and specific entity. Giving particular attention to study design and statistical analysis, we identify a large and robust gene expression profile of 2959 genes that discriminates DSS paediatric patients from other dengue patients, DF or DHF, who did not progress to shock, whatever the supportive treatment they received. Importantly, DSS children clustered together whatever they were considered as having primary or secondary dengue infection, while secondary infections represented the majority of DF, DHF and DSS children recruited (see table S1), as expected in hyper-endemic areas. The robustness of the DSS-associated gene signature was established by showing that the disease phenotype variable significantly affected expression levels of all the genes identified (multi-way ANOVA) and demonstrating the classifying capability of this gene signature using unsupervised hierarchical clustering and SVM/leave-one-out methods [35], [39], [40]. Based on unsupervised hierarchical clustering, DHF grades I/II patients' expression profiles appear very close or indistinguishable from those of DF patients at the same time of disease evolution, while they group into two heterogeneous sub-groups (1 and 2b, Figure 1), of which significance should be investigated. Altogether, the present results highlight the inadequacy of the 1997 WHO classification of dengue clinical forms [19]–[21], that considers DF and DHF grades I/II as two separate disease phenotypes, and support the recently proposed classification [90].

Two important questions arise about the DSS-associated transcriptional profile: are the observed modifications of genes expression the cause or the consequence of the pathology, and could these modifications have a predictive value? We cannot definitively answer these questions from the present study, in part because blood samples were collected at the onset of shock (14 out of the 19 DSS patients) or after (5 patients). Functional study of each individual pathway will be required to fully understand the role of each gene in a complex network of molecular interactions.

The ability of some genes transcripts or genes products to accurately predict progression to DSS should be evaluated by multivariate regression models [91] using blood samples collected before the onset of shock, while this proves to be difficult in the context of dengue outbreaks [28]. In the present study, we chose to focus on those of the identified molecular mechanisms that made the more sense to DSS pathophysiology and systemic vascular dysfunction, referring to recent findings on the role of innate immunity in systemic inflammatory processes leading to shock, multi-organ dysfunction syndromes or other pejorative clinical outcomes.

Second, while present results confirm some putative DSS-related biomarkers, it also reveals unreported alterations that make sense to hypovolemic shock pathophysiology. This reinforces the ability of a global and “open mind” approach to identify molecular processes relevant to the studied pathology. Blood cells transcriptional profiles clearly reveal alterations of different immune responses and the activation of a large pro-inflammatory response. A significant proportion of genes of which expression is modified are related to host innate immunity, lymphocyte functions and lipid metabolism in particular. This genome-wide expression analysis also confirms the over-expression of individual biomarkers previously associated with severe dengue, such as the acute phase pentraxin-related protein PTX3, the pro-inflammatory IL-18 cytokine or the anti-inflammatory IL-10 cytokine (Table S2) [11], [12], [15], providing a more comprehensive overview of their implication in the pathophysiology of DSS.

Our results differ however from those reported by Long *et al* in a genome-wide expression profiling study comparing DSS children with uncomplicated paediatric patients [28]. This study concluded on a global “benign” and “muted” immune transcriptional response but a decreased expression of genes involved in IL-10 and IFN type I-related pathways in DSS children blood cells [28]. Differences in study design, size of cohorts and time of blood sampling from patients in the course of dengue disease may explain these differences. Indeed, in our study, two DSS children had gene expression profiles close to those of uncomplicated DF and DHF, and clustered within the DF/DHF cluster. Both proved to be the children from whom blood was sampled three days after the onset of shock, while the three DSS children sampled two days after shock onset still exhibited a typical DSS gene expression profile. This suggests that a shift from a “severe” to an “uncomplicated” transcriptional profile may occur within a very short time, and could explain the “uncomplicated” and benign gene immune transcriptional responses reported by Long *et al* [28]. Differences in strategies and methods used to filter genes differentially expressed between patients' groups could also explain the finding that few IFN type I-related genes but a large diversity of other pathways were identified in the present study compared to other transcriptomic studies of DHF or DSS patients. Here, genes were selected considering only their statistical significance and their association with the disease phenotype. Differently from others [27], [28], [42], no “fold change” cut-off filter was applied since this non-statistically-motivated criteria selects preferentially genes prompt to high variations such as the IFN type I-induced genes [92], [93], thus excluding from subsequent bio-informatic analysis a diversity of transcripts exhibiting more subtle variations but strong associations and biological relevance with the considered disease phenotype.

Third, unsuspected mechanisms identified in DSS patients could contribute importantly to the pathophysiology of this severe syndrome, as supported by similarities between those DSS-related alterations and other critical syndromes. Interestingly, a number of immune, repair-remodeling and metabolic-related related pathways are simultaneously altered in the blood cells of DSS children at the onset of shock. In particular, T and NK lymphocyte transcriptional responses are globally impaired while genes implicated in compensatory anti-inflammatory and repair/remodeling responses and in innate immune responses are over-expressed. This highlights the complexity of biological responses at the time of dengue shock syndrome, and points out similarities between DSS and other critical syndromes such as severe sepsis, or post-trauma SIRS that are similarly characterized by depressed T lymphocyte responses but over-expressed innate immunity [94], [95].

Reduced abundance of a number of T-lymphocyte related transcripts at the time of DSS may reflect a feed-back mechanism aimed at limiting an initial early T lymphocyte activation, reported to occur in patients who further progress to severe dengue [96], [97]. Such a negative feed-back may be sustained by the over-expression of a diversity of anti-inflammatory transcripts in DSS patients' blood cells at the time of shock. In particular, the two potent immunomodulating factors prostaglandin E synthase and VSIG4, which dampen both T and NK lymphocyte responses [43]–[45] and have both a strong statistical association with the DSS phenotype, could have such a negative effect. Based on those observations and previous clinical reports, the benefit of corticotherapy in DSS patients might thus be questioned [98], [99].

Over-expression in the blood of DSS children of several repair and remodeling genes encoding extracellular matrix proteins, vasoactive mediators and matrix metalloproteases such as the MMP9, likely reflects a compensatory response to inflammatory insults, and a number of those genes products are now considered putative biomarkers in systemic inflammatory syndromes such as severe sepsis [100]. Most proteins encoded by those genes are indeed secreted by activated immune cells such as monocytes/macrophages. They may have adverse effects towards the vascular endothelium when produced in excess, since they may increase immobilization of inflammatory mediators at the surface of endothelial cells [101], permeability of capillaries [102], or induce direct damage to endothelial tissues [103]. Recently, one of them, MMP9 has been proposed as a putative candidate in the occurrence of plasma leakage during dengue infection [47].

While previous transcriptional studies failed to identify pro-inflammatory gene patterns in the blood cells of DSS patients [27], [28], [42], our study is the first one to report that a diversity of pro-inflammatory transcriptional responses at the interface of innate immunity, inflammation and host lipid metabolism are activated at the time of cardiovascular failure. Since those mechanisms are considered pathogenic in other systemic inflammatory diseases where systemic vascular dysfunction does occur, we suggest that they may altogether contribute to DSS pathophysiology.

Activation of a pro-inflammatory defence gene pattern in DSS patients' blood cells (Table 4) has relevance to the pathophysiology of systemic vascular dysfunction, since most microbicidal peptides and enzymes have recognized pro-inflammatory and pathogenic effects towards vascular endothelial tissues [50]. Among them, the neutrophil microbicidal peptides alpha defensins and the highly pro-inflammatory calgranulins proteins S100A8/A9 and S100A12 are now considered putative pathogenic factors in sepsis, cardiovascular diseases, rheumatoid arthritis or atherosclerosis [51], [56], [104]. While neutrophils are considered the main source of those defence molecules, this cellular origin cannot be established from the present study due to the cellular complexity of unfractionnated whole blood samples and to the possibility that other circulating cell types may express a neutrophil-like inflammatory repertoire under pathologic conditions [105]. A putative neutrophil origin of this gene expression pattern is however supported by the over-expression in DSS patients blood cells of transcripts encoding other neutrophil-related molecules such as the MMP8 matrix metalloprotease, CEACAM-6, CEACAM-8 and CD99L2 adhesion molecules (Tables 4 and S2) involved in the recruitment of neutrophils to vascular endothelia. Functional studies should confirm whether those first line-defence immune cells which produce an array of pro-inflammatory mediators highly damaging to host tissues and vascular endothelia [50], [106], and poorly regarded in dengue [107], definitively play a role in DSS pathophysiology.

Alteration of a gene pattern related to homeostasis of cholesterol in monocytes/macrophages (Mo/Mac) in the blood cells of DSS children (Table 5) was an unexpected finding, while it should be considered regarding recent knowledge on the role of monocytes as a pivotal link between inflammation, innate immunity and host lipid metabolism [108], [109]. Indeed, under physiological conditions, monocytes maintain cholesterol homeostasis by clearing modified LDL such as oxidized LDL (ox-LDL) from plasma. Under pathological conditions, balance between uptake and efflux of those modified cholesterol molecules may be altered [59], [60], [62], [66], [67], [86], resulting in the intracellular accumulation of modified cholesterol. This turns classical monocytes towards a pro-inflammatory phenotype, lipid-laden monocytes/macrophages (Mo/Mac), a sub-type of pro-inflammatory immune cells initially identified in vascular lesions of chronic inflammatory metabolic diseases [110].

Recent knowledge has shown that those atypical monocytes produce a large array of pro-inflammatory mediators such as ROS, metalloproteases, eicosanoids, and pro-inflammatory adipokines, making these cells potent contributors to vascular damages, systemic inflammation and major metabolic changes such as insulin-resistance [109], [111], which altogether characterize systemic inflammatory syndromes such as DSS or severe sepsis. While the existence of functional lipid-laden Mo/Mac during DSS should be established by functional studies, such a molecular mechanism could explain decrease of circulating sub-fractions and total cholesterol previously reported in DSS [112]–[115] and in other critically ill patients where low cholesterol levels are associated with poor clinical outcome [116], [117].

Altered homeostasis of cholesterol in blood cells from DSS patients could also favour replication of dengue viruses into host cells [118], thus contributing to increased viremia in patients with severe dengue infection [119], [120], while this could not be evaluated in this study since part of patients had undetectable viremia at the time of blood sampling.

The factors contributing to altered homeostasis of cholesterol in the blood cells of DSS children at time of shock are numerous. Increased lipid peroxydation activity [113], [114] insufficiently compensated by anti-oxydant mechanisms [121], as supported by related altered gene patterns identified in this study (Table 5), may result in high levels of circulating ox-LDL contributing to altered cholesterol metabolism. Differences in nutritional status [122]–[124] or host genetics may also contribute to altered homeostasis of cholesterol gene pattern in the blood cells of DSS patients. Interestingly, transcripts encoding molecules considered candidates to diseases characterized by impairment of cholesterol homeostasis such as *NPC1*, *PCSK9* and *PPARG* [66], [67], [74], have significant altered abundance in the blood cells of DSS children (Table 5). Further investigations should consider possible associations between DSS and allelic variants of such genes. Whatever the determinants of cholesterol metabolism alterations in DSS patients, our results reinforce interest in considering sub-fractions and total cholesterol as putative biomarkers of DSS [115]. They also suggest that drugs used to treat metabolic disorders such as atherosclerosis should deserve further attention for the control of such a pro-inflammatory process in dengue-infected patients, now proposed for other critical illnesses [117].

Transcriptional activation of the lipid-related arachidonic acid pathway in the whole blood cells of DSS children at the time of shock was another pro-inflammatory mechanism relevant to the pathophysiology of DSS [81]. Activation of this lipid metabolic pathway in innate cells such as neutrophils or lipid-laden monocytes during inflammatory process or infection [125], results in the production of eicosanoid lipid mediators, that are not only physiological regulators of vascular tone and permeability [81] but also potent pro-inflammatory mediators involved in a number of pathologies such as asthma [81]. Interestingly, formation of lipid bodies where eicosanoid synthesis takes place, can be induced by ox-LDL through activation of the PPARγ nuclear-lipid receptor [126], thus suggesting a direct link between the three pro-inflammatory pathways identified in DSS children and a contribution of arachidonic pathway-related inflammatory lipids and oxidative enzymes to the systemic vascular dysfunction leading to DSS.

Fourth, DAMPs and TLRs could be a link from primary to secondary inflammation, leading to DSS. Occurrence of DSS in only some patients at the late phase of infection is likely due to an inadequate control or an amplification of the primary inflammatory response aimed at fighting infection. The pro-inflammatory molecular responses activated in the blood cells of DSS children at time of shock involve a diversity of innate immune mediators that may amplify a first-line inflammatory response mediated by TNF, IL-6 or IL-1, thus contributing to a secondary inflammatory loop. Indeed, a number of repair/remodeling and of defence gene products over-expressed in DSS patients blood cells are considered endogenous danger signals or Danger-Associated Molecular Patterns (DAMPs) (Tables 3 and 4; molecules with DAMP activity are indicated) capable to trigger secondary systemic inflammatory responses through direct interaction with surface or intracellular receptors such as TLRs or NODs expressed in endothelial or innate immune cells [127]. DAMPs include a diversity of molecules without structural similarity either actively produced by immune cells in the context of an infection or passively secreted by damaged tissues [128], [129], now considered key inducers of secondary systemic inflammation in a number of acute inflammatory syndromes [130], [131] or chronic diseases [132].

Amplification of inflammation during DSS through direct signalling by molecules harbouring DAMP activity via TLRs, is also supported by the increased abundance of DAMP-induced transcripts as those encoding the pro-inflammatory IL-18 cytokine or the NLRC4/CARD12 intracellular sensor [55]. Interestingly, association of allelic polymorphisms of TLR4 with DSS suggested by De Kruif and colleagues [46] suggests that differential signalling through TLRs may contribute to the severity of dengue disease, as suspected for other pathologies [133]. Accordingly, anti-inflammatory drugs targeting Toll-like receptors are now under development for a number of inflammatory pathologies where innate immunity and TLRs play a central pathogenic role [134].

### DSS pathophysiology: a secondary inflammatory loop hypothesis

To summarize, we report the identification of a specific gene expression profile in the blood cells of DSS children at time of shock, characterizing DSS as a unique entity at the transcriptional level whatever the immunological status of children regarding primary or secondary infection. Major immunological alterations identified at the time of shock are characterized by an altered balance between depressed T lymphocyte responses and exacerbated compensatory and pro-inflammatory innate immune responses that may, finally, be detrimental to the host [135]–[137], while functional studies should confirm the contribution of those molecular mechanisms to DSS pathophysiology.

**Figure 4:**
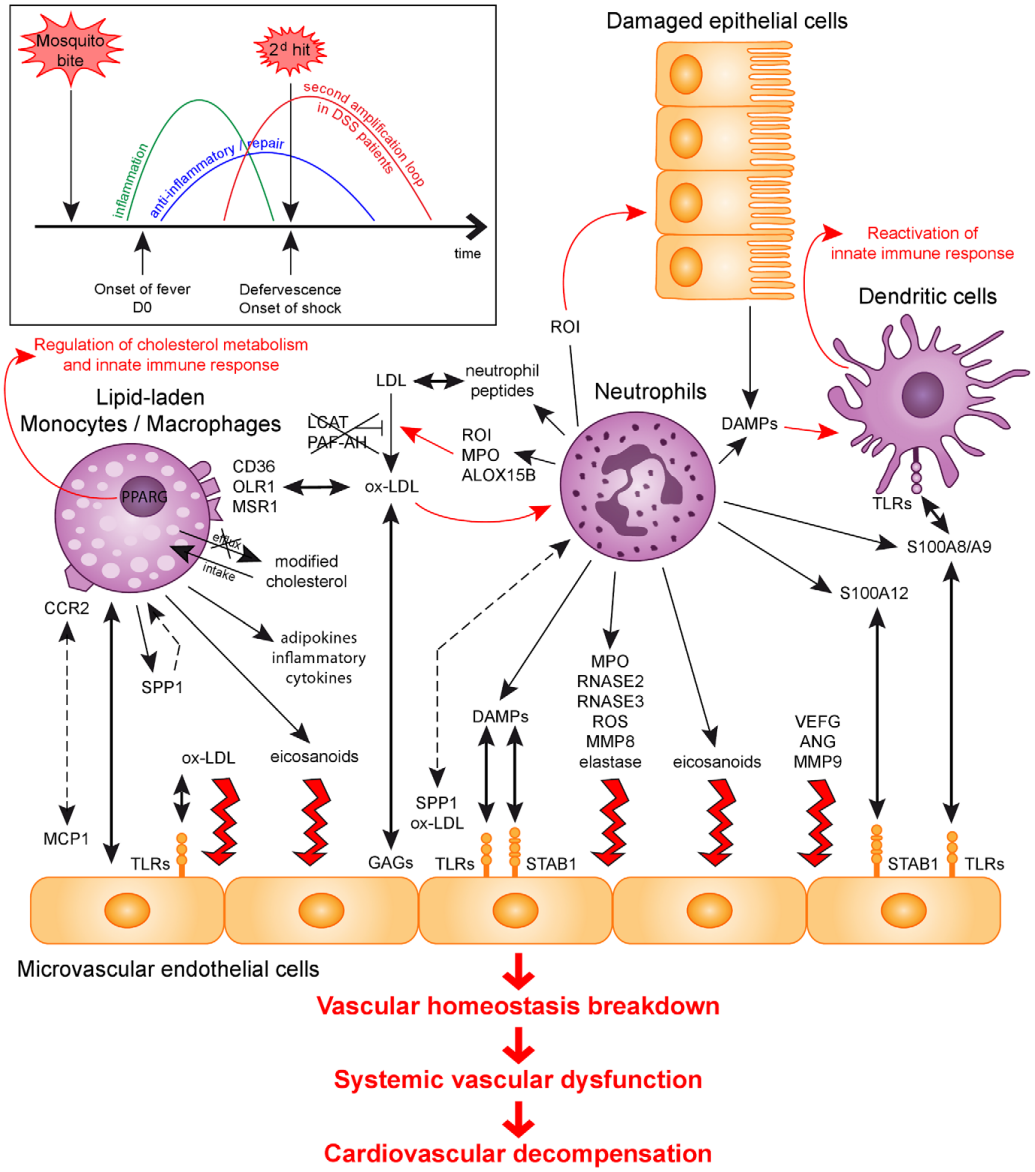
Hypothesis of a second inflammatory amplification loop in dengue shock syndrome. After induction of a first inflammatory and anti-viral response to dengue virus, disease resolution generally occurs around time of defervescence for most dengue-infected patients. Some patients however progress towards a life-threatening dengue shock syndrome. Results obtained in this study suggest that in those patients, a second inflammatory amplification loop, which involves a diversity of pro-inflammatory responses related to innate immunity, occurs and leads to a major inflammatory systemic syndrome and to vascular homeostasis breakdown. The putative role of different markers identified in vascular endothelial dysfunction is indicated. Thin black arrow, release of; Bold black arrow, interaction between; Punctuated black arrow, chemotactic effect; Thin red arrow, biological activity; Bold red arrow, direct activity on endothelium. DAMPs, danger-associated molecular pattern; GAG, glycosaminoglycane; ROI, reactive oxygen intermediates; TLR, Toll-like receptor.

Based on recent knowledge on molecular mechanisms altered in other systemic inflammatory diseases, DSS may result from a complex pro-inflammatory network involving a diversity of innate immune effectors sustaining a secondary systemic inflammatory loop, leading in turn to vascular homeostasis breakdown and systemic microcirculatory failure characterizing DSS (Figure 4).

We suggest that drugs available to treat metabolic and other systemic chronic inflammatory diseases could be considered for the treatment of dengue-infected patients before shock occurs, and that a number of bio-markers found altered in DSS patients blood cells should be evaluated. as putative predictive markers of progression to DSS.

## Supporting Information

Figure S1 Validation of microarray results by RT-PCR. Pearson's correlation was calculated between microarray expression signals (horizontal axis) and Delta Ct values from real-time PCR (vertical axis) for nine genes highly associated to dengue shock syndrome. ** Correlation is significant at 0.01.
(4.94 MB TIF)

Table S1 Clinical and biological characteristics of each DF, DHF and DSS patient
(0.04 MB XLS)

Table S2 List of the 3515 clones corresponding to the 2959 genes differentially expressed between DF, DHF and DSS patients, identified using the multi-way ANOVA at a false discovery rate of 10. Clones corresponding to the 2959 genes are listed according to their association to DSS, the first one being the gene of which expression level variance is the most influenced by the clinical phenotype. HUGO gene names are indicated. The variation is the one related to the DSS group relatively to DF and DHF. ANOVA, analysis of variance; DF, dengue fever; DHF, dengue hemorrhagic fever; DSS, dengue shock syndrome; NA, not available. a percentage of variance associated to disease phenotype.
(0.90 MB XLS)
