# Supplementary figures and images for: Genome-Wide Expression Profiling Deciphers Host Responses Altered during Dengue Shock Syndrome and Reveals the Role of Innate Immunity in Severe Dengue
Source: PLoS One. 2010 Jul 20;5(7):e11671. doi: 10.1371/journal.pone.0011671 (PMC2907396; doi:10.1371/journal.pone.0011671)

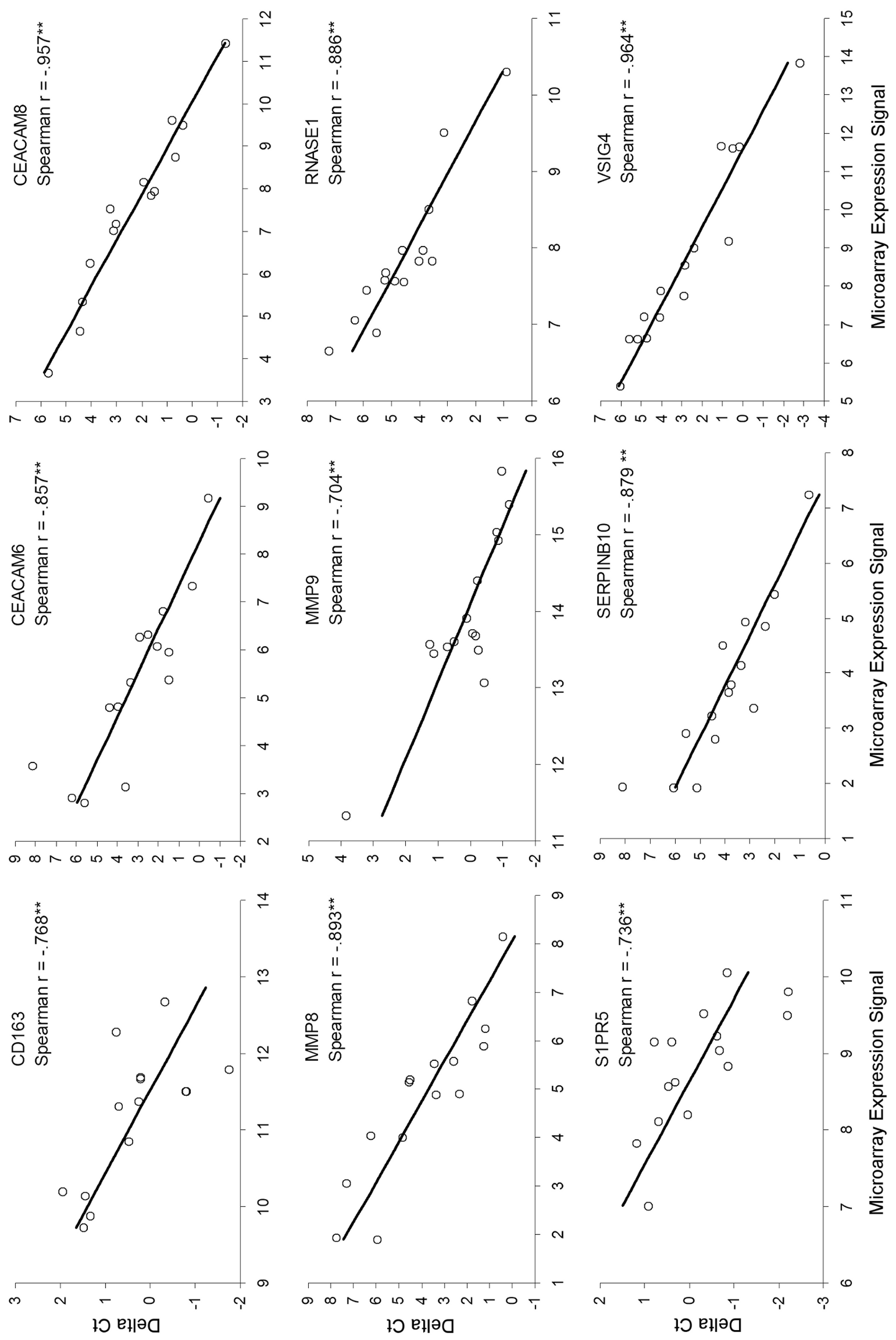

Supplement: Figure S1 — Validation of microarray results by RT-PCR. Pearson's correlation was calculated between microarray expression signals (horizontal axis) and Delta Ct values from real-time PCR (vertical axis) for nine genes highly associated to dengue shock syndrome. ** Correlation is significant at 0.01. (4.94 MB TIF) [file pone.0011671.s001.tif]
